# Policy Choices for Progressive Realization of Universal Health Coverage: Comment on "Ethical Perspective: Five Unacceptable Trade-offs on the Path to Universal Health Coverage"

**DOI:** 10.15171/ijhpm.2016.99

**Published:** 2016-07-31

**Authors:** Viroj Tangcharoensathien, Walaiporn Patcharanarumol, Warisa Panichkriangkrai, Angkana Sommanustweechai

**Affiliations:** International Health Policy Program, Ministry of Public Health, Nonthaburi, Thailand.

**Keywords:** Progressive Realization of Universal Health Coverage (UHC), Equity and Efficiency Trade-off, Political Choices, Thailand

## Abstract

In responses to Norheim’s editorial, this commentary offers reflections from Thailand, how the five unacceptable trade-offs were applied to the universal health coverage (UHC) reforms between 1975 and 2002 when the whole 64 million people were covered by one of the three public health insurance systems. This commentary aims to generate global discussions on how best UHC can be gradually achieved. Not only the proposed five discrete trade-offs within each dimension, there are also trade-offs between the three dimensions of UHC such as population coverage, service coverage and cost coverage. Findings from Thai UHC show that equity is applied for the population coverage extension, when the low income households and the informal sector were the priority population groups for coverage extension by different prepayment schemes in 1975 and 1984, respectively. With an exception of public sector employees who were historically covered as part of fringe benefits were covered well before the poor. The private sector employees were covered last in 1990. Historically, Thailand applied a comprehensive benefit package where a few items are excluded using the negative list; until there was improved capacities on technology assessment that cost-effectiveness are used for the inclusion of new interventions into the benefit package. Not only cost-effectiveness, but long term budget impact, equity and ethical considerations are taken into account. Cost coverage is mostly determined by the fiscal capacities. Close ended budget with mix of provider payment methods are used as a tool for trade-off service coverage and financial risk protection. Introducing copayment in the context of fee-for-service can be harmful to beneficiaries due to supplier induced demands, inefficiency and unpredictable out of pocket payment by households. UHC achieves favorable outcomes as it was implemented when there was a full geographical coverage of primary healthcare coverage in all districts and sub-districts after three decade of health infrastructure investment and health workforce development since 1980s. The legacy of targeting population group by different prepayment mechanisms, leading to fragmentation, discrepancies and inequity across schemes, can be rectified by harmonization at the early phase when these schemes were introduced. Robust public accountability and participation mechanisms are recommended when deciding the UHC strategy.

## Background

In Norheim’s editorial,^1^ based on fairness, equity and ethical grounds, his five “unacceptable trade-offs” in implementing universal health coverage (UHC) seems convincing. Our analysis found that three out of these five are related to equity goal; trade-off II (prioritize those who are able to pay than the poor and informal sector), trade-off IV (prioritize the well-off than the worse-off) and trade-off V (move from out of pocket payment by households to a less progressive mandatory prepayment source of finance).

The remaining two are related to efficiency goal: trade-off I (extend coverage to the low or medium priority services than the high priority services) and trade-off III (provide costly services with low health benefits than less costly high impact services). Note that the unacceptable trade-off II and IV are very close. In this editorial, efficiency is regarded as part of fairness.

Since health resources are finite, using it for one purpose, policy-makers have to sacrifice other alternates; hence trade-off and priority setting is unavoidable. These unacceptable trade-offs are theoretically sound and convincing; they are useful caveats for which policy-makers in low- and middle-income countries (LMICs) may use to make informed, fair and ethical choices in their paths towards UHC.

This commentary offers reflections from a country experiences, Thailand, how these trade-offs were (or were not) applied in implementing UHC reforms since 1975 until UHC was reached in 2002,^2^ and achieved favourable outcomes.^3^ This commentary aims to generate global discussions on how best UHC can be gradually achieved.

## UHC Cube: Trade-off Within and Across Three Dimensions

**Figure 1:**
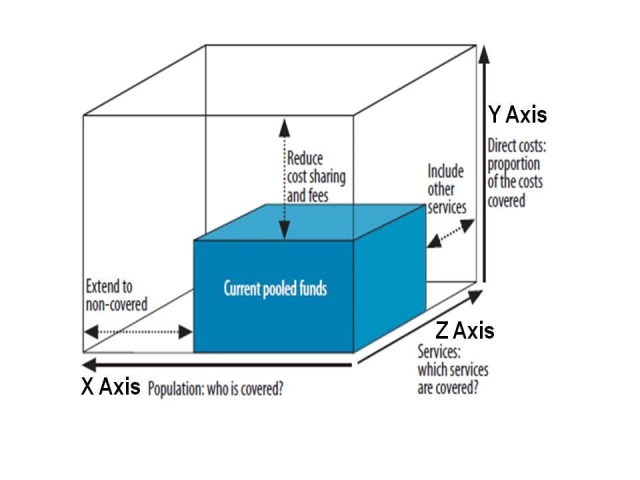
The Three Dimensions of WHO UHC Cube. Abbreviations: WHO, World Health Organization; UHC, universal health coverage.

Trade-off is a situation where one must decide to choose between or balance the two alternatives that are opposite or cannot be taken at the same time. There are three dimensions of the UHC cube (see Figure 1). The X axis is the population coverage, the Y axis is the cost coverage measured by level of out of pocket cost sharing by members, and the Z axis is the service coverage, how comprehensive the benefit package would cover? There are also trade-offs between these three dimensions such as should the country cover more services to certain groups, or same service for the whole population?

## X Axis: Population Coverage

Within each of the three dimensions, there are trade-offs. On the X axis, we concur with Norheim’s assertion, on an ethical ground, that the poor, the worse off and certain disadvantaged groups who are unable to pay their medical bills should be covered first. This ethical choice will gain high political support, if these population sub-groups are vocal constituencies who cast their votes or influence others in an election every four to five years in developing countries. Unfortunately, very often they are voiceless and powerless.

Increasingly, private sector employment are growing especially in middle-income context, that payroll tax financed social health insurance (SHI) systems should be introduced as soon as possible, in order to minimize the regressive out of pocket payment, with a caveat that payroll tax finance must be designed as a progressive source, where the higher income employees pay higher contribution than the lower income counterparts. When window of opportunities open, SHI can be introduced immediately, and no need to wait for full coverage of the poor.

In developing countries, implementing tax financed scheme dedicated for the poor, or exempting them from paying user fees is challenging. Thailand medical welfare scheme for the low income households introduced in 1975; premium funded voluntary health card scheme for the informal sector in 1984; and payroll tax financed SHI scheme for private sector employees in 1990 demonstrated the explicit political decision on population extension on the X axis based on ethical principles, the more vulnerable they are, the higher priority they receive.

## Z Axis: Service Coverage

On the Z axis, which service package is offered to different population group is a political choice, often governed by the government fiscal spaces and how priority is made; either informed by cost-effectiveness evidence, financial risk protection, equity or pragmatism. Often there is limited technology assessment capacity in developing countries. Though global evidence is available such as Disease Control Priority^4^ and Cost-effectiveness and strategic planning (WHO-CHOICE),^5^ countries need capacities to translate them into policies and implementation. As comprehensive benefit package was fully applied in all financial risk protection schemes, it is not possible to apply a new positive list covering basic essential package; hence pragmatism is applied by Thai reform, with the application of negative list, where all services are covered except a few in the list.

Despite the cost-ineffectiveness and large budget impact, renal replacement therapy for kidney failure patients, a life threatening condition, was approved by the government in 2008 on equity ground and financial protection. Two other schemes, the government employee and the private sector employee schemes have full coverage of renal replacement treatment; should not the universal coverage scheme (UCS) get this similar service? Cost of treatment is catastrophic to UCS members, certain patients died from inadequate out of pocket financed dialysis, leaving behind a large debt to repay by family.^6-8^

Inequity arises when certain services are not available in remote rural areas where the poor live, but enjoyed by urban rich population. Introducing UHC without adequate and equitable distribution of supply side capacity is prone to pro-rich outcomes, as demonstrated in China^9^ and Philippines.^10^ While extensive geographical coverage of functioning primary healthcare determines the pro-poor UHC outcomes.^11,12^

The Thai UHC was introduced after three decades of government investment in health service infrastructure in particular district health systems, and ensuring functioning of health service through mandatory rural services by health professional graduates.^13^ Skilled birth attendance had reached 99.3% of total births; and contraceptive prevalence 79.2% of women age 15-49 in 2000, well before UHC achievement in 2002.^14^ An extensive geographical coverage of functioning health services is the foundation for effective UHC implementation.

## Y Axis: Cost Coverage

On Y axis, cost sharing is interlinked with X axis, which population group should or should not co-pay, and interlink with Z axis, which services should be fully subsidized. Clearly, Thailand applied equity principle where the poor are exempted from payment or copayment; and efficiency principle where services such as maternal and child health, immunization, cost effective interventions and community-based public health interventions are fully subsidized to the whole population, not only the poor due to external benefits. Until recently when UHC was achieved in 2002 that all services in the benefit package are fully covered, free at point of services; this is not because improved fiscal capacity but the application of close end payment which has the merits of cost containment and system efficiency. Efficiency frees up more resources for zero co-pays.

A caveat on copayment, introducing copayment as percentage of the medical bills in particular when insurance agency applies fee-for-service is harmful to the patients in particular the low income; ample evidence shows that fee-for-service stimulates supplier induced demand (demand in excess of what patient would choose) because of information asymmetry in healthcare market, hence professional acts as patients’ agent and making decision on their behalf. Fee-for-service provides opportunities for professionals to maximize services.^15^ Copayment can be applied to discourage bypassing primary healthcare. However, ensuring quality at primary healthcare to gain citizens’ trust and confidence are important prerequisites.

Strategic purchasing comes into play to contain cost and protect members from catastrophic spending and medical impoverishment. Institutional capacities to manage purchasing by insurance agencies are contributing factors to efficiency and equity.^3^ Cost coverage in Y and service coverage in Z axes are interlinked under the strategic purchasing design and implementation.^13^

UHC achieves favorable outcomes as it was implemented when there was a full geographical coverage of primary healthcare coverage in all districts and sub-districts after three decade of health infrastructure investment and health workforce development since 1980s.

## Thailand UHC Trajectory: A Long March Between 1975 and 2002

At the bottom layer, people living below national poverty line was covered by publicly financed medical welfare schemes, launched in 1975, which gradually extended to cover all elderly, children under 12 years old, persons with disability and village health volunteers.

At the top layer, government employees and their dependents are historically covered by non-contributed tax financed scheme, as part of the comprehensive welfare. Civil servants’ salary is claimed to be lower than labour market. The private sector employees are covered by payroll tax financed SHI, launched in 1990, as part of the comprehensive social security including pensions and unemployment benefits.

The informal sector, at the middle layer, was covered by voluntary premium financed public insurance launched in 1984 by the Ministry of Public Health, and later 50% of premium was subsidized by the government in 1992. Despite Ministry of Public Health’s efforts, coverage remained low; by 2001, 30% of total population was uninsured. Clearly, voluntary nature of prepayment scheme cannot achieve UHC.

**Figure 2:**
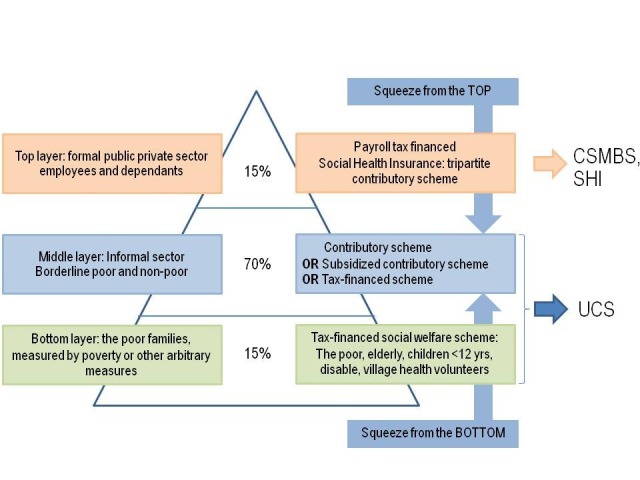
Thailand Trajectories Towards UHC. Abbreviation: UHC, universal health coverage.

In 2002, in keeping UHC political manifesto in the 2001 general election, decisive political decision was made to cover the whole bottom and middle layers by UCS, financed by general tax. Tax is one of the most progressive sources of financing.^3^ Squeezing from the bottom described in Figure 2, by tax financed reflects strong government commitment on UHC. It is technically not feasible to enforce premium payment by the large size informal sector and their irregular income; while premium financed UCS is political non-palatable. Effectiveness of premium collection and equity in financial contribution were the two main concerns in extending coverage to the informal sector. Additional budget required to finance UCS is within fiscal capacities in 2002. The use of closed end budget with mixed provider payment methods in UCS contains cost and prevents supplier induced demands.

Arguments arise on inequity in financial contribution that private sector employees have double contributions: payroll-tax for SHI coverage and general tax (direct and indirect) while UCS members only contribute to general tax. This may incentivize an increased informality in the economy. Tax financed UHC is the political choice and societal preference to maintain payroll tax financed SHI. It is a political correct and technical sound decisions to apply tax-finance UHC in 2002 when Thailand decided to achieve UHC fairly quickly.^16^ Advocates to abolish contribution in SHI is not a political correct and economic sound proposal given the increased size of formal sector.

Finally, the reform in 2002 resulted in UCS for the bottom and middle layers, 75% of total people; while keeping intact the Civil Servant Medical Benefit Scheme for government employees and SHI for private employees. The application of close ended budget facilitates more comprehensive service coverage and high financial risk protection for UCS and SHI members.

The key designs contributing to favorable outcomes are; a comprehensive benefit package and free at point of service contributes to high level of financial risk protection as measured by the incidence of catastrophic health expenditure and medical impoverishment^3^; contracting with district health systems contributes to pro-poor use of services and public resources as measured by benefit incidence.^11^

## After UHC: Fragmentation Across Different Schemes

Fragmented schemes are essential feature of UHC transition when most countries apply targeting population groups. Norheim assumes a single entity in making decisions about how to expand coverage; there are many actors having stakes on UHC, such as Ministry of Health, Ministry of Labour and also private insurance agencies. Expansions of services that are privately financed are hard to influence in a laissez fair economy.

In the stride towards UHC, various countries extend coverage to different population sub-group when windows of opportunity opened, for example the poor subsidized by general tax, the private sector employees by SHI payroll tax financed scheme, and informal sector by premium financed voluntary community based health insurance, with or without government subsidies, such as the case of Thailand,^2^ China,^17^ Lao PDR.^18^ These trajectories result in discrepancies across different insurance schemes in term benefit package and provider payment — causing inequity and inefficiency.

In the paths towards UHC, LMICs should recognize these challenges facing the pathfinder countries; efforts should be given to minimize the gap of inequity through harmonization strategic purchasing (in particular benefit package, level of public subsidies and provider payment methods) across different schemes, if unavoidably different schemes for different population groups are applied.

## Conclusion

We fully support Norheim’s recommendation that “Robust public accountability and participation mechanisms are, therefore, essential when deciding on the overall strategy and the appropriateness of central trade-offs on the path to UHC.” However, not all LMICs have such platform. Cross country learning and sharing lessons from UHC pathfinder countries convened by international development partners, as well as institutional capacity strengthening focusing on strategic purchasing function are further recommended.

Expansions of financial risk protection are incremental processes where there is no “clean slate” furnished with all ethical options for making UHC choices; reformists should stand ready when the political windows open to re-orient toward more equitable and ethical choices.

## Acknowledgements

We pay tributes to the late professor Nikom Chantaravitura and Dr. Sanguan Nittayaramphong, who are the champions in marshalling Thailand adoption of Social Security Scheme in 1990 and Universal Coverage Scheme in 2002. We acknowledge policy-makers, managers, program implementers, front line health workers and researchers for their contributions in the design, implementation, monitoring, and evaluation of UHC.

## Ethical issues

Not applicable.

## Competing interests

Authors declare that they have no competing interests.

## Authors’ contributions

VT conceptualizes and starts the first draft. All authors contribute to strengthening the manuscript, reviewed and signed off the final version.
